# Evaluation and Management of Intrauterine Device (IUD) Complications in Ethiopia

**DOI:** 10.1002/puh2.70162

**Published:** 2025-10-24

**Authors:** Abraham Fessehaye Sium, Amani Nureddin Abdu, Sarah Prager

**Affiliations:** ^1^ Department of Obstetrics and Gynecology St. Paul's Hospital Millennium Medical College (SPHMMC) Addis Ababa Ethiopia; ^2^ Division of Complex Family Planning Department of Obstetrics and Gynecology UW Medicine Seattle Washington USA

**Keywords:** complex family planning, Ethiopia, failed IUD, IUD removal, low‐income country, missing IUD, uterine perforation

## Abstract

**Background:**

Though safe in most circumstances, intrauterine device (IUD) has some rare serious complications such as missing IUD, uterine perforation, missing strings, and pregnancy with IUD in situ (failed IUD). This study reviewed IUD complications and management techniques utilized at a national complex family planning center in Ethiopia.

**Methods:**

This was a retrospective study of women who had an IUD complication and were managed at St. Paul's Hospital Millennium Medical College in Addis Ababa, Ethiopia between May 2017 and April 2024. Data were collected retrospectively by reviewing the medical records of patients. Data were analyzed using SPSS version 23. Simple descriptive statistics were employed. Frequency and proportions were used to present the results.

**Results:**

Thirteen cases were excluded due to incomplete data. Out of the 27 women included in the final analysis, 12/27 (44.4%) had a missing IUD, followed by 7/27 (25.4%) who had missing strings. There were two cases of pregnancy with IUD in situ. Among those with a missing IUD, 10 were diagnosed with uterine perforation and were managed surgically (9 managed laparoscopically and one managed with an open laparotomy). Both women with a pregnancy and IUD in situ chose to continue the pregnancy after removing the IUD.

**Conclusion:**

In this study, missing IUD (with uterine perforation diagnosed in 10 out of 12 patients) was the most common complication, occurring in close to half of the study participants. Ultrasound should be routine in evaluation of missing IUDs, and laparoscopy should be considered the gold standard for extrauterine removal, even in low‐resource settings (patients with such serious complication should be referred to centers that have this capacity).

## Graphical abstract

Missing IUDs should be carefully evaluated with ultrasound, as uterine perforation is a common finding. We support laparoscopic removal of IUD as the standard for management for extrauterine IUDs.

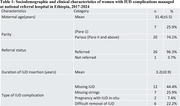

## 1 Introduction

Currently, strong evidence shows that long‐acting reversible contraception (intrauterine devices [IUDs] and implants) is superior at preventing pregnancy (20 times more effective) to that of short‐acting contraceptives such as pills, patch, or ring and can be used safely by most women throughout the reproductive lifetime [1, 2, 3]. Though the IUD is the most widely used reversible method of contraception in the world, with as much as 14.5% utilization in developing countries [4], IUD use as a contraceptive method in Ethiopia remains low. According to EDHS 2019, IUD use in Ethiopia is less than 2% [5].

Despite being safe in most circumstances, IUD has some serious complications such as missing IUD strings (only indicating a problem with the strings, which may have just retracted into the cervix or uterus), missing IUD (IUD device gone from the uterus, potentially due to unnoticed expulsion or uterine perforation), and IUD migration. When IUD fails, pregnancy with IUD in situ can also occur [6]. Very rarely, complications of IUD can lead to mortality or serious morbidity. According to earlier reports published five decades ago, the mortality attributable to IUDs is estimated to be lower than that attributable to combination oral contraceptives. In one case series from the United States, five maternal deaths were reported, three due to sepsis and two resulting from failed IUDs and complications from subsequent pregnancy [7]. In 2009, one case series documented four patients with serious intraperitoneal sepsis over an 18‐month interval. Each was associated with long‐term retention of a copper IUD in the peritoneum, which was identified as the likely source of infection [8].

The increasing uptake of IUDs is making complications more evident, which, in turn, makes diagnosis and management of the rare IUD complications far more important. Understanding and communicating these risks to clients during contraceptive counseling is essential, and it encourages providers to take appropriate precautions during IUD insertion, as the timing of most of these complications is associated with insertion. Moreover, it helps to identify complications early and manage them when they inevitably occur [9].

Rare complications of IUD insertion, like uterine perforation, are well described in international literature. However, there is inadequate data regarding its management in low‐income settings, and current evidence on this issue in these settings is mainly based on case reports. For example, in Ethiopia, there is one case report that documented a case of a 27‐year‐old woman who presented with missing IUD strings and was diagnosed with uterine perforation, managed via laparotomy for IUD removal [10]. Similarly, another case report published by Ahmed et al. presented one case of missing IUD with migration into the abdominal cavity managed with open surgery [11]. Recently, following the launch of a family planning fellowship training in Ethiopia at St. Paul's Hospital Millennium Medical College (SPHMMC), the majority of patients with rare IUD complications are managed laparoscopically, including those referred from other facilities. Our study reviewed cases of IUD complications managed at this national family planning excellence center in Ethiopia between 2017 and 2024.

## 2 Methods

This is a retrospective study of women with an IUD complication who were managed at SPHMMC between May 2017 and April 2024. SPHMMC is a leading national referral hospital, which hosts a large number of referred patients from different regions of Ethiopia who require tertiary‐level evaluation and intervention. The college runs the only active family planning fellowship training program in Africa, and MICHU clinic within the college is the site for the practical component of this training as well as overall comprehensive sexual and reproductive health (SRH) service. This has helped advance the quality and extent of comprehensive abortion care and family planning services, including standard management of IUD complications. The primary objective of this study was to describe the frequency and management techniques used for less common IUD complications, such as missing IUDs, missing strings, pregnancy with IUDs at this tertiary hospital.

Data were collected retrospectively by reviewing the paper‐based patient medical records. We used a structured data extraction form to obtain the data from medical records. Sociodemographic characteristics (maternal age), reproductive characteristics (parity), and clinical characteristics of IUD complication and management were collected. The inclusion criteria were IUD complications assessed and managed at the MICHU clinic, the family planning clinic at SPHMMC. Those with incomplete information, including management techniques, were excluded. Maternal age, parity, and clinical characteristics of IUD complication and management (referral status, duration of IUD insertion, and complication type) were collected.

No sample size calculation was employed. Data were analyzed using SPSS version 23. Simple descriptive statistics were applied to analyze the data. Frequency and percentage were used to present the results. Formal ethical clearance letter was obtained from SPHMMC IRB. The study was exempted from the requirement for obtaining informed consent from the study subjects.

## 3 Results

There were 220 women who presented for IUD removal during the study period, out of whom 40 women had an IUD complication at the time of presentation. After excluding 13 patients for missing data, we included 27 women with an IUD complication into the final analysis. The mean age of the participants was 31.4 (±5.5) years (Table 1). Most of them were parous women who had postpartum IUD insertion, with almost three‐quarters (20/27, 74.1%) having at least two children. Almost all the study participants were referred from other centers, with only one patient (3.7%) who had IUD insertion at our hospital. The mean duration of IUD insertion among the women was 3.2 (±0.9) years. The most common IUD complication was missing IUD (12/27, 44.4%), followed by missing string (7/27, 25.9%). In all cases of missing strings, the IUD was localized with ultrasound within the uterus. Pregnancy with IUD in situ was diagnosed in two women (7.4%).

**TABLE 1:** Sociodemographic and clinical characteristics of women with intrauterine device (IUD) complications managed at national referral hospital in Ethiopia, 2017–2024.

Characteristics	Category	*n*	%
Maternal age (years)	Mean	31.4 (±5.5)	
Parity	(Para‐1)	7	25.9
	Multi‐parous (Para‐II and above)	20	74.1
Referral status	Referred	26	96.3
	Not referred	1	3.7
Duration of IUD insertion (years)	Mean	3.2 (±0.9)	
Type of IUD complication	Missing IUD	12	44.4
	Missing strings	7	25.9
	Pregnancy with IUD in situ	2	7.4
	Difficult removal of IUD	6	22.2

Among the missing IUD cases, 10 of them were diagnosed with uterine perforation and managed surgically, whereas two patients with missing IUDs seven patients with missing strings, and six cases referred to as difficult IUD removal cases were successfully managed with IUD removal using alligator forceps (Table 2). Among those who presented with missing IUDs, 9 (33.3% out of the total 27) had uterine perforation and had their IUDs removed laparoscopically, whereas one woman had her IUD removed via open laparotomy. For both women pregnant with an IUD in situ, the IUD was removed with a plan to continue the pregnancy. One patient subsequently returned a week later with a miscarriage, and the second patient had a live term birth.

**TABLE 2:** Characteristics and outcome of intrauterine device (IUD) management techniques utilized in the management of women with IUD complications at national referral hospital in Ethiopia, 2017–2024, n = 27.

Characteristics	Category	*n*	%
Management technique of IUD complication	Difficult removal with alligator forceps (6 difficult removal of IUD, 7 missing strings, and 2 ruled out missing IUD)	15	55.6
	Laparoscopic removal of IUD (missing IUD)	9	33.3
	Open laparotomy and removal of IUD (Missing IUD)	1	3.7
	Pregnancy continued with IUD in place (IUD in pregnancy)	2	7.4
Complication encountered during IUD removal	None	27	100

## 4 Discussion

In this study of women who presented for complicated IUD removal at our center, missing IUD (with uterine perforation diagnosed in 10 out of 12 patients) was the most common complication, occurring in close to half of the study participants. This is followed by missing IUD strings, diagnosed in a quarter of women included in the study. Nine out of the 10 women with uterine perforation had their IUD removed laparoscopically.

IUD expulsion, contraceptive failure (pregnancy with IUD in situ or increased risk of ectopic pregnancy), uterine perforation, and misplaced IUDs are the most important complications from IUD use [12]. Providers’ lack of experience, anatomical disorders of the uterus and the cervix, breastfeeding, and improper timing of IUD placement are also the most notable risk factors of uterine perforation during IUD insertion [13]. The importance of pelvic ultrasound in monitoring after IUD insertion (correct and safe insertion), as well as in decreasing and identifying complications of IUD, is well‐documented in multiple literature [14, 15, 16, 17]. Although rare, IUD migration can be encountered that requires multidisciplinary team management approach [18].

Uterine perforation at the time of IUD placement is uncommon, with an incidence of 0.8–2.2 per 1000 IUD insertions. Beyond adhesion formation in the peritoneum, very rarely it can lead to colonic or bladder perforations [19, 20, 21]. Mild abdominal pain in combination with missing IUD on pelvic exam is the most common clinical presentation [22]. The recommended management of extrauterine IUD (IUD with uterine perforation) is by laparoscopy [23]. In our study, all the 10 women who had uterine perforation were referred as missing IUD, and nine of them had their IUDs removed laparoscopically, with only one patient who had IUD removal via laparotomy. Missing IUD strings [24] are an uncommon finding, typically resulting in confirmation of appropriate intrauterine placement of the IUD with no evidence of extrauterine IUD. A study of 14,935 IUD users identified 750 (5.0%) users who presented with missing IUD strings at any follow‐up visit. Ultrasound ultimately confirmed an intrauterine IUD in 735 of them (98.0%) [25]. In this study, missing strings was the second most common type of IUD complication encountered, and all seven of the women had their IUDs localized within the uterine cavity using ultrasound and removed transcervically using alligator forceps.

There is some debate over the best management of an IUD in situ with a desired pregnancy [26]. Current level‐I evidence shows that continuing pregnancy with IUD in place increases the risk of spontaneous and induced abortions by up to 7–23 fold, respectively [27, 28]. IUD removal and continuation of pregnancy reduces, but does not entirely eliminate, pregnancy risks [28, 29]. Another systematic review of nine articles showed that early IUD removal appeared to improve outcomes but did not eliminate risks. In this study, there were two women who had desired pregnancy with IUD in situ. In both cases, the IUD was removed using alligator forceps, and pregnancy continued. One of them ended up miscarrying during the first trimester, whereas the second one continued the pregnancy and achieved a live birth at term. We underscore cautious interpretation of the outcomes related to pregnancies with IUD in situ in the present study, given the small number of cases.

This study is one of the first studies on IUD complications and management in a low‐income, specifically Sub‐Saharan, setting. Findings of our study, including those related to management characteristics, imply the need for expansion of complex family inform training programs in Ethiopia and similar contexts so that patients with such complications receive the highest standard of management timely. In one word, it indicates the need for reforming national family planning policy in low–middle‐income countries in favor of wider and easy access for such complex family planning services.

Lack of analysis of true incidence of IUD complications among all insertions is the main limitation of this study. Our hospital is a referral center for complex family planning cases, and many of the patients in this study had their IUD inserted at another facility, limiting our ability to determine a true denominator of all IUD insertions—incidence rates cannot be derived from this study due to lack of denominator data. The other limitation of this study is small sample size. We recommend a country‐wide, prospective, multicenter study, in which case the IUD complications with the denominator of total insertions could be determined.

## 5 Conclusion

Our study shows that missing IUDs should be carefully evaluated, as uterine perforation was a common finding. We support laparoscopic removal of IUD as the standard for management for extrauterine IUDs. Our study supports careful ultrasound evaluation of missing IUDs and missing strings, as uterine perforation was diagnosed in the majority of cases, whereas confirmation of IUD in situ was made in all of the cases in the latter. We underscore ultrasound as a critical evaluation tool, even in low‐income settings.

## Author Contributions

Abraham Fessehaye contributed conception and development of the study design. Abraham Fessehaye and Amani Nureddin Abdu contributed data collection and data analysis. Abraham Fessehaye and Sarah Prager contributed manuscript writeup. The final manuscript was edited by Sarah Prager. All authors critically revised the article for intellectual content. All authors reviewed the final manuscript and approved its submission.

## Funding

The authors have nothing to report.

## Ethics Statement

Formal ethical clearance letter was obtained from Institutional Review Board of St. Paul Hospital Millennium Medical College.

## Consent

Informed consent was not needed for this study, so it was not obtained from study participants.

## Conflicts of Interest

Abraham Fessehaye is an editorial board member at Public Health Challenges, and he was excluded from the editorial decision‐making for this article. All the other authors declare no conflicts of interest.

## Data Availability

The data that support the findings of this study are available on request from the corresponding author. The data are not publicly available due to privacy or ethical restrictions.
